# Quantitative dynamics of *Salmonella* and *E*. *coli* in feces of feedlot cattle treated with ceftiofur and chlortetracycline

**DOI:** 10.1371/journal.pone.0225697

**Published:** 2019-12-02

**Authors:** Naomi Ohta, Bo Norby, Guy H. Loneragan, Javier Vinasco, Henk C. den Bakker, Sara D. Lawhon, Keri N. Norman, Harvey M. Scott

**Affiliations:** 1 Department of Veterinary Pathobiology, College of Veterinary Medicine and Biomedical Sciences, Texas A&M University, College Station, Texas, United States of America; 2 Department of Large Animal Clinical Sciences, College of Veterinary Medicine, Michigan State University, East Lansing, Michigan, United States of America; 3 School of Veterinary Medicine, Texas Tech University, Amarillo, Texas, United States of America; 4 Center for Food Safety, University of Georgia, Griffin, Georgia, United States of America; 5 Department of Veterinary Integrative Biosciences, College of Veterinary Medicine and Biomedical Sciences, Texas A&M University, College Station, Texas, United States of America; Cornell University, UNITED STATES

## Abstract

Antibiotic use in beef cattle is a risk factor for the expansion of antimicrobial-resistant *Salmonella* populations. However, actual changes in the quantity of *Salmonella* in cattle feces following antibiotic use have not been investigated. Previously, we observed an overall reduction in *Salmonella* prevalence in cattle feces associated with both ceftiofur crystalline-free acid (CCFA) and chlortetracycline (CTC) use; however, during the same time frame the prevalence of multidrug-resistant *Salmonella* increased. The purpose of this analysis was to quantify the dynamics of *Salmonella* using colony counting (via a spiral-plating method) and hydrolysis probe-based qPCR (TaqMan^®^ qPCR). Additionally, we quantified antibiotic-resistant *Salmonella* by plating to agar containing antibiotics at Clinical & Laboratory Standards Institute breakpoint concentrations. Cattle were randomly assigned to 4 treatment groups across 16 pens in 2 replicates consisting of 88 cattle each. Fecal samples from Days 0, 4, 8, 14, 20, and 26 were subjected to quantification assays. Duplicate qPCR assays targeting the *Salmonella invA* gene were performed on total community DNA for 1,040 samples. Diluted fecal samples were spiral plated on plain Brilliant Green Agar (BGA) and BGA with ceftriaxone (4 μg/ml) or tetracycline (16 μg/ml). For comparison purposes, indicator non-type-specific (NTS) *E*. *coli* were also quantified by direct spiral plating. Quantity of NTS *E*. *coli* and *Salmonella* significantly decreased immediately following CCFA treatment. CTC treatment further decreased the quantity of *Salmonella* but not NTS *E*. *coli*. Effects of antibiotics on the imputed log_10_ quantity of *Salmonella* were analyzed via a multi-level mixed linear regression model. The *invA* gene copies decreased with CCFA treatment by approximately 2 log_10_ gene copies/g feces and remained low following additional CTC treatment. The quantities of tetracycline or ceftriaxone-resistant *Salmonella* were approximately 4 log_10_ CFU/g feces; however, most of the samples were under the quantification limit. The results of this study demonstrate that antibiotic use decreases the overall quantity of *Salmonella* in cattle feces in the short term; however, the overall quantities of antimicrobial-resistant NTS *E*. *coli* and *Salmonella* tend to remain at a constant level throughout.

## Introduction

Salmonellosis is one of the most common foodborne diseases in the United States [1]. Detection of *Salmonella* in retail ground beef has been low (0.4–2%) in reports from the National Antimicrobial Resistance Monitoring System (NARMS) from 2002 to 2015 [2]. However, retail ground beef is one of the primary sources of foodborne *Salmonella* infection for humans in the United States and multiple outbreaks have been reported due to consumption of under-cooked ground beef [2, 3]. The finishing period for cattle occurs in the feedlot, and this is where *Salmonella* can readily spread via the fecal-oral route or the environment (e.g., feed and flies) among infected and uninfected cattle residing in the same and adjacent pens [4, 5]. Most often, cattle are asymptomatic carriers and no clinical signs are shown.

*Salmonella* contamination of meat mainly occurs at the slaughterhouse through aerosolization of fecal contaminated hides onto carcasses and from fat-shrouded lymph nodes containing *Salmonella* being incorporated into batches of ground beef [6–11]. Several studies have shown higher prevalence of *Salmonella* on hides than in feces at slaughter; therefore, hides and lymph-nodes are likely sources of carcass and ground beef product contamination, respectively [9, 12, 13]. Contamination of meat with antibiotic-resistant *Salmonella* poses a serious public health threat due to there being few treatment options for high-risk populations such as infants, children, pregnant women, and immunocompromised persons [14].

In lieu of fluoroquinolones, which may cause musculoskeletal disorders, cephalosporins are the choice of treatment for the high-risk populations listed above. Approximately 80% of cephalosporins that are sold for use in food animals are used in cattle production [15]. Since *Salmonella* is not considered an adulterant in raw meat by the United States Department of Agriculture, it is crucial to decrease both overall *Salmonella* carriage and unnecessary antibiotic use at the feedlot. This will ultimately reduce hide and carcass contamination of *Salmonella* overall, and especially of antibiotic-resistant *Salmonella* at slaughter. Ideally, such reductions will extend to lymph nodes as well. The effects of antibiotic use on the quantities of antibiotic-resistant *Salmonella* have not been studied systematically in feedlot cattle.

Both chlortetracycline (CTC) and the third-generation cephalosporin, ceftiofur, are antibiotics used for the treatment and control of bovine respiratory diseases (BRD) in feedlot cattle. In our previous work, we demonstrated that the prevalence of *Salmonella* decreased immediately during and following ceftiofur and CTC treatment; however, the proportion of multidrug resistant (MDR) *Salmonella* also increased [16]. One-time treatment of cattle with ceftiofur had a continuous effect on elevating the prevalence of MDR *Salmonella* populations until the study terminated after 26 days. The CTC treatment had and even stronger effect on increasing the proportion of MDR *Salmonella* than did the ceftiofur treatment. From a quantitative perspective, however, it remained unclear whether the depleted susceptible *Salmonella* populations were replaced by resistant *Salmonella*, or the quantity of resistant *Salmonella* stayed the same. In the current study, we have quantified both the susceptible and resistant *Salmonella* populations in order to clarify the effects of antibiotics.

The effects of antibiotic use on the quantity of *E*. *coli* in cattle have previously been studied [17–20]. Most studies of *E*. *coli* have demonstrated that antibiotic treatment decreases overall *E*. *coli* concentrations transiently [17]. However, the change in the quantity of antibiotic-resistant *E*. *coli* has not been clearly demonstrated. Data on absolute and relative changes in resistant bacteria following antibiotic treatments are necessary for quantitative risk assessment analysis for foodborne antibiotic-resistant bacteria [21]. Therefore, in the current study, we quantified *E*. *coli* in addition to *Salmonella*.

As a continuation from our previous research concerning the prevalence of *Salmonella* (i.e., absence/presence and serotype), the goal of the present study was to explore the quantitative population dynamics of resistant and susceptible bacteria following antibiotic treatment in cattle. The quantity of *Salmonella* present in a sample–versus its mere presence or absence–is especially relevant for risk assessments as the risk of human foodborne salmonellosis is dose-dependent [22]. Tetracycline and ceftiofur/ceftriaxone were chosen as the antibiotics of primary interest, since resistance to tetracycline is common among both *E*. *coli* and *Salmonella* strains in feedlot cattle and often presents as co-resistance to cephalosporins [23–26].

## Materials and methods

### Experimental design and sample collection

The experimental design was the same as described in previous work by this group [16, 26]. Briefly, we conducted a randomized controlled longitudinal field trial during two sequential 26-day replicates in an experimental feedlot at West Texas A&M University in Canyon, Texas, USA. A high prevalence of *Salmonella* in cattle is commonly observed in this area [16]. The first replicate was started in early August and a second replicate in the middle of September of 2009. The cattle were purchased from a single operation and were shipped directly to the experimental feed yard one month before the trial started. It is unknown if the cattle had previously been assembled at the source. The animals were yearling steers that were predominantly of the Angus breed and were fed diets typical of regional feedlots; that is, a flaked-corned based diet with added roughage, protein, vitamins and minerals. If any cattle became sick and required antimicrobial treatments, they were excluded from the study. In each replicate, 88 steers were assigned into 8 pens (n = 11 cattle) to distribute the body weights among the pens evenly in a two-by-two factorial design with four treatment regimens, as described previously [16, 26]. Four pens were assigned into each treatment group among two replicates. Across both replicates, all 11 steers received 6.6 mg/kg of CCFA (EXCEDE^®^, Zoetis Animal Health, Florham Park, NJ) treatment subcutaneously at the base of the ear in 8 pens (described as “All-CCFA & CTC” and “All-CCFA/no CTC” in this article; group metaphylaxis model), and in the remaining 8 pens, a single steer treated with CCFA on day 0 was co-housed (mixed) with 10 non-treated steers (S1 Fig). A single steer was mixed in the pen to reproduce a real feedlot situation, such that one steer is treated with CCFA for BRD and send back to its original pen. Repeated within each of the two replicates, four of the pens assigned CCFA treatments received 22 mg/kg CTC (Aureomycin^®^, chlortetracycline complex equivalent to 220.5 g/kg of chlortetracycline, Alpharma, Bridgewater, NJ). The CTC was top-dressed in feed for five consecutive days over three time periods with a one-day interval in between. The CTC feeding occurred during the period from day 4 until day 20 (S1 Fig: “All-CCFA & CTC”, “1-CCFA & CTC”). The remaining 8 pens in each replicate did not receive CTC (S1 Fig: “All-CCFA/no CTC” and “1-CCFA/no CTC”). Fecal samples were collected every other day *per rectum* and samples from days 0, 4, 8, 14, 20, and 26 were tested [16, 26]. Fecal samples from Day 0 were collected before the treatment with CCFA and considered as baseline. Samples for culture were mixed with glycerol at a 1:1 ratio and preserved at −80 °C; samples for qPCR analysis were similarly preserved but without glycerol.

The sample size of this study was calculated based on the expected quantities of *E*. *coli* and not with *Salmonella*, since the original study was designed to investigate antibiotic resistant *E*. *coli* in the cattle population, where it could be estimated as 100%. *Salmonella* prevalence were estimated to be much smaller than that; unexpectedly, the prevalence was much higher at 70%. This meant 30% of counts were below the limit of detection which posed problems for the variance estimates and assumptions of normal residuals and was part of the motivation for the imputation approach, given we did not believe the true quantity was zero for all negative samples. That said, with a log10 transformed mean of 6 and 4 for *E*. *coli* and *Salmonella*, respectively, and accounting for pen- and animal-level dependencies (intracluster coefficients) of .05 and .10 for log10 CFU, respectively, we estimated a sample size per group of 16 (cluster adjusted to 40) would provide a power of 0.8 and 95% confidence for a 0.5 log10 difference. This calculation outcome does not depend on the mean of 4 versus 3.5 (*Salmonella*) or 6 versus 5.5 (*E*. *coli*).

The animal experiments were approved by the Amarillo-Area Cooperative Research, Education, and Extension Triangle Animal Care and Use Committee (Protocol No. 2008–07), and by the Clinical Research Review Committee at Texas A&M University (CRRC # 09–35). All experiments were performed in accordance with institutional and United States Department of Agriculture (USDA) guidelines and regulations governing the oversight and conduct of experiments involving food producing animals. Institutional Biosafety Committee approval # IBC 2014–043 at Texas A&M University permitted the microbiological laboratory experiments involving *Salmonella enterica* serotypes and *E*. *coli*.

### Quantification of *Salmonella* and NTS *E*. *coli* by colony counting

Fecal samples (500 mg) from days 0, 4, 8, 14, 20, and 26 of replicates 1 and 2 were diluted in 4.5 ml of phosphate buffered saline (PBS) at a 1:9 ratio. Diluted fecal samples were plated on BGA, BGA with 16 μg/ml of tetracycline (BGA-tet), and BGA with 4 μg/ml of ceftriaxone (BGA-cef) using an Eddy Jet^®^ 2 spiral plater (Neutec Group Inc, Farmingdale, NY) with the E-Mode 50 μl setting and incubated at 37 °C for 18 hours. The concentrations of antibiotics were chosen following CLSI breakpoints for tetracycline and ceftriaxone, respectively [27]. Presumptive *Salmonella* colonies were counted by an automated Flash & Go^®^ colony counter the next day following the manufacturer’s instructions (Neutec Group Inc, Farmingdale, NY).

Fecal samples also were diluted and spiral plated on MacConkey agar (MAC), MAC with tetracycline (16 μg/ml), ceftiofur (8 μg/ml), and both drugs (16 and 8 μg/ml, respectively) for NTS *E*. *coli* quantification. The concentration of ceftiofur was chosen following NARMS consensus breakpoints [28]. Previous work had shown that 99.9% of isolates grown on MAC agar from cattle feces were confirmed as NTS *E*. *coli* [29]. The plates were incubated for 18–24 hrs, colonies were counted and then back-calculated through dilution factors to CFU/g feces.

### Hydrolysis probe (TaqMan^®^ probe) quantitative real-time PCR (probe-qPCR) for *Salmonella*

#### Total community DNA extraction for qPCR

Total community DNA was extracted from 200mg of non-glycerol-diluted feces from days 0, 4, 8, 14, 20, and 26 of replicate 1 and 2 by the QIAamp DNA Stool Mini Kit ^™^ (Qiagen, Valencia, CA) in the QIAcube robot ^™^ (Qiagen, Valencia, CA) following the manufacturer’s instructions as described previously [30]. The quality and quantity of the community DNA was estimated via the NanoDrop^®^ ND-1000 UV-Vis Spectrophotometer (NanoDrop Technologies, Wilmington, DE) at wavelengths of 260 and 280 nm. DNA samples were stored at -20 °C for further genotypic analysis.

#### Standard curve generation

The genomic DNA extracted from *Salmonella enterica* subsp. *enterica* serovar Typhimurium ATCC^®^ 700720^™^ (ATCC^®^, Manassas, Virginia) using the QIAcube ^™^ robot (Qiagen, Valencia, CA) was used as a template for standard curve generation [31]. The DNA sample was serially diluted, and each standard curve reaction contained 3 μl of diluted genomic DNA, in which the final copy numbers were 1x10^6^, 1x10^5^, 1x10^4^, 1x10^3^, 1x10^2^, and 1x10^1^. Gene copy numbers were estimated following the calculation described previously [32]. The total size of the *Salmonella* Typhimurium ATCC 700720 strain is 4,857,450bp (GenBank: AE006468.2). The molar mass per base pair was set as 650 (g/mol)/bp. Efficiency was calculated following the equation:

$$\text{efficiency}=10^{\left(-1/\text{slope}\;\text{of}\;\text{standard}\;\text{curve}\right)}-1$$

#### Primers/probes and reactions setup

Standard curve generation and the primers and probes targeting the *invA* gene, carried by all *Salmonella* and necessary for invasion of intestinal epithelial cells [33, 34], were adapted and modified from Gonzalez-Escalona et al. (Table 1) [35]. FAM was used for the 5’-Reporter dye (Table 1). The 16s rRNA data were utilized from a previous study (Table 1) [30, 36]. Primer specificity was confirmed by primerBLAST, which returned only *Salmonella enterica* subsp. *enterica*. The number of *invA* gene copies in *Salmonella* serotypes found in the study population was previously reported to be a single gene copy per genome [16, 31]. Quantities were standardized via the 16s rRNA quantity in the community DNA as described previously [30].

**Table 1:** Primers and probes used for qPCR.

Gene	Primer	Sequence	Tm (°C)	Product size (bp)	Genbank accession number
*invA*	*invA*_176_F	5’-CAA CGT TTC CTG CGG TAC TGT-3’	60	116	M90846
	*invA*_291_R	5’-CCC GAA CGT GGC GAT AAT T -3’			
	*invA*_FAM_208 Probe	FAM-CTC TTT CGT CTG GCA TTA TCG ATC AGT ACC A-Iowa Black RQ-Sp			
16S rRNA	*1056F*	5′—AAT GTT GGG TTA AGT CCC GCA ACG—3′		400^1^	EU014689
	*1456R*	5′—ATG ATC ACA AAG TGG TAA GCG CCC—3′			
	*P201*	5′- GAG GAA GGI GIG GAI GAC GT—3′		216^2^	
	*P1370*	5′—AGI CCC GIG AAC GTA TTC AC—3′			

^1^Broad range primers to generate template for the standard curve [30]

^2^Narrow range primers for sample quantification [30]

Each reaction was composed of 10 μl of Brilliant III Ultra-Fast QPCR Master Mix with Low ROX (Agilent Technologies, Santa Clara, California), 4.6 μl of nuclease-free water, 0.4 μl of probe (0.2 μM), 1 μl each of forward and reverse primers (0.5 μM), and 3 μl of total community DNA. Thermocycler conditions for the reaction were 95°C for 3 min (activation of master mix), and 40 cycles of 95°C for 5 sec (denaturing), 60°C for 10 sec. (annealing/extension) on AriaMx Real-Time PCR system (Agilent Technologies, Santa Clara, California) [31, 35, 37]. Data collection for fluorescence was conducted at the annealing/extension step of each cycle. The reaction plates were set up manually and each sample was run in duplicate. The assays were subjected to qPCR performance analysis following the MIQE guidelines for qPCR [38].

## Probe-qPCR data analysis

After each run, qPCR data were analyzed using the AriaMx ver. 1.0 software (Agilent, Santa Clara, CA). Gene copy numbers were back-calculated to gene copies per gram of wet feces by taking feces loss and dilutions into account at each step of community DNA extraction. Cq derived gene copy numbers were multiplied by 583.33 for the complete back calculation considering the sample lost during DNA extraction process. Both non-standardized *invA* gene copies per gram wet feces and the estimates obtained by standardizing with the 16S rRNA gene were calculated and analyzed.

### Statistical analysis

#### Analysis of qPCR and colony count data using multilevel mixed-linear regression

All standardized, non-standardized, and colony count derived quantities (CFU/g feces wet matter) were transformed to log base 10 for use as dependent variables in multiple imputation and mixed-linear regression models performed with Stata/IC 14.2 (StataCorp LLC, College Station, TX). Counts from direct plating on plain agar and antibiotic-containing agar for both *Salmonella* and NTS *E*. *coli* were log_10_ transformed and used for dependent variables in multilevel mixed-linear regression. CCFA pen mixing (binary), CTC (binary), and days (integer) were treated as independent fixed factor variables in a 3-way full factorial model. Replicate, pen, and animal ID were potential clustering variables and included as random effects. qPCR derived log_10_ transformed outcomes were rounded to the nearest whole number and converted from continuous numeric to integer data for the purpose of analysis; further, since many of the qPCR reactions were below the limit-of-quantification (LOQ) these were marked as zero. These counts were set as dependent variables, and CCFA (binary), CTC (binary), and days (integer) were included as independent fixed factor variables specified in a 3-way full factorial model. 1-CCFA mixing (0), No-CTC treatment (0), and day 0 were set as the baseline. The same clustering variables as above were used as inflation variables. The log_10_ transformed counts were used for linear regression analyses of both qPCR and spiral plating.

#### Imputation of missing values

When the estimated quantity of the *invA* gene was under the LOQ they were recorded as missing values. The assumption was that samples containing *Salmonella* at concentrations less than the LOQ were unlikely to truly harbor zero bacteria. Originally, missing values were given a value of 1 and log_10_ transformed to 0 for visualization purposes. The missing values were explored by imputation procedures in Stata/IC 14.2 with linear regression, truncated regression, and interval-censored regression to provide a censored continuous variable [39, 40]. Each imputation was conducted with the full factorial model including trial replicate as a fixed effect, and a 3-way full factorial model using CCFA, CTC, and day as independent variables. The upper limit of missing values was set as 3.6 log_10_ (mean and median of the *invA* gene copy numbers in the samples with one-missing qPCR value) for those with missing observations in truncated regression and interval-censored regression. The lower limit was set to -∞. The imputed values were diagnosed for fit with the observed data [40]. A representative imputed value set was chosen for multilevel mixed-linear regression to assess the effects of antibiotic treatments by day on the quantity of *Salmonella* represented by *invA* gene copies per gram of feces. In Stata, estimation following multiple imputation can be performed; however, no programmed methods are provided to predict margins from the estimation model. Margins of treatment effects on log_10_ *invA* gene copies per gram feces and those standardized with 16s were predicted by multilevel mixed linear regression.

## Results

### Summary statistics of direct spiral plating and qPCR

A total of 386 (37.1%) samples were quantifiable for *Salmonella* using either spiral plating, qPCR, or both among all tested samples (S2(a) Fig). More samples (n = 324) were quantifiable by qPCR than direct plating (n = 252) (S2(a) Fig). Most of the samples that grew on antibiotic-containing agars (e.g., BGA-tet, and BGA-cef) had growth on BGA-plain; unexpectedly, 5 of these samples did not grow on BGA-plain (S2(b) Fig).

The LOQ was 2.6 log_10_ per gram feces for direct spiral plating and 2.78 log_10_ per gram feces for qPCR (Table 2). The 16s rRNA gene was quantifiable in all samples.

**Table 2:** Summary of quantification in direct spiral plating and probe qPCR.

Organism	Quantifying method	Quantifiable	Below LOQ	Min-Max (log_10_/g feces)	Mean (log_10_/g feces)
*Salmonella*	Direct spiral plating	252 (24.2%)	788 (75.7%)	2.60–6.18	3.54 (95% CI: 3.44–3.63)
	Probe qPCR	324 (31.2%)	716 (68.8%)	2.78–7.99	4.33 (95% CI: 4.24–4.42)

**Fig 1:**
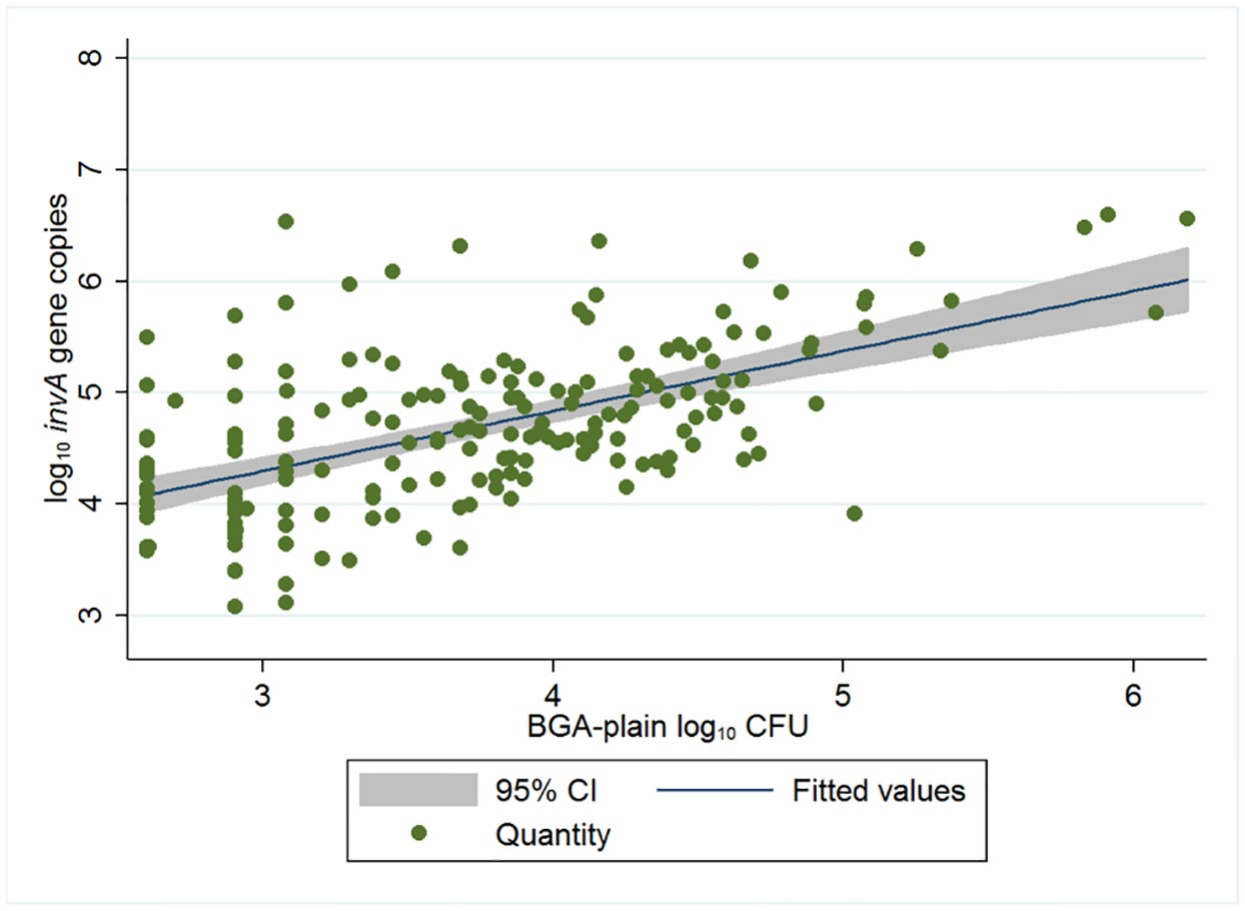
Linear prediction line and scatter plot of quantities of log_10_ *invA* genes via qPCR and log_10_ CFU on BGA-plain agar. Only with samples quantified using both methods (n = 190). The R^2^ was 0.43, the coefficient was 0.54 and the intercept was 0.61.

Linear regression for log_10_ *invA* gene copies based on the log_10_ CFU counts was strongly correlated (p < 0.0001) (Fig 1). The mean quantity was higher in qPCR than on BGA-plain, and the difference between those was 0.96 log_10_ (data points ranged from -1.13 to 3.46). Zero values in qPCR were imputed in the later analyses, since it was suspected that many of them were below LOQ, but not actually zero.

### Quantitative dynamics of *Salmonella* in cattle treated with CCFA and CTC

**Fig 2:**
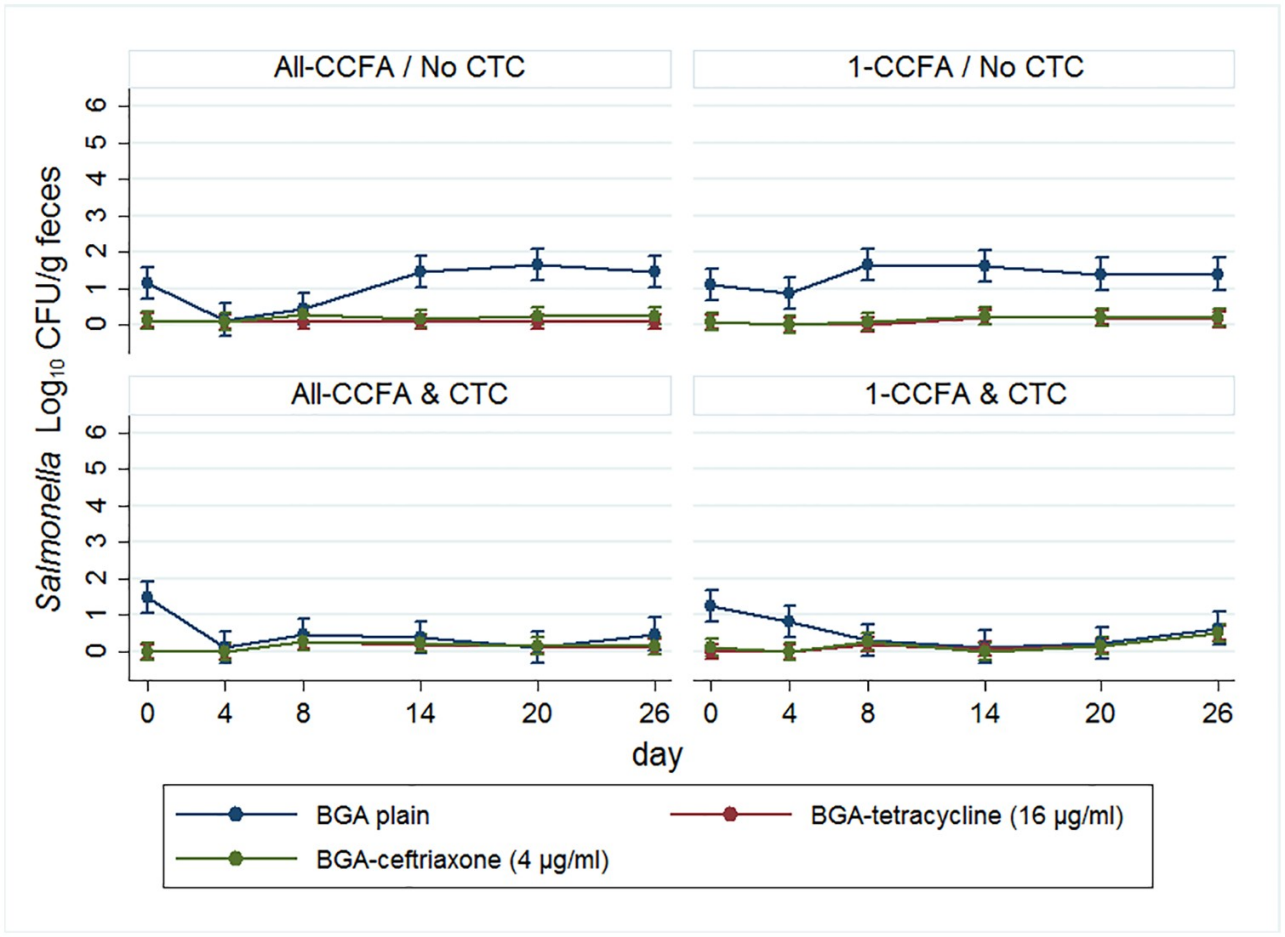
Quantity of *Salmonella* modeled using multilevel mixed linear regression. Blue: colony growth on plain BGA, red: colony growth on BGA-tetracycline, green: colony growth on BGA-ceftriaxone.

*Salmonella* counts on plain BGA agar modeled using multi-level mixed linear regression are presented in Fig 2. CCFA treatment (All-CCFA group) significantly decreased the quantity of *Salmonella* at Day 4 (p = 0.037) and Day 8 (p < 0.0001). Without additional CTC treatments, the *Salmonella* quantity recovered to initial levels by Day 14 (All-CCFA / No CTC). With additional CTC treatments following CCFA, the quantity of total *Salmonella* remained significantly lower than No CTC on Days 14, 20, and 26 (p < 0.0001 in all days) (All-CCFA & CTC group). Similarly, CTC treatment by itself (1-CCFA & CTC group) decreased the quantity of *Salmonella*; however, absent the earlier treatment with CCFA the quantity recovered near to the initial level by Day 26 (Fig 2).

In the 1-CCFA & No-CTC group, cattle shedding ceftriaxone- and tetracycline-resistant *Salmonella* over the LOQ increased by Day 26 which is potentially troubling as it represents a change from the baseline day 0 values in the model. Therefore, observed data of resistant (and susceptible) *Salmonella* on BGA-cef and BGA-tet are shown in S3 Fig. Although not statistically significant, counts of ceftriaxone-resistant *Salmonella* increased on Day 8 following the CCFA treatment in the All-CCFA groups and tetracycline-resistant *Salmonella* also were impacted by the additional tetracycline treatments (All-CCFA & CTC) (S3 Fig). Perhaps surprisingly, CTC treatments appeared to have greater effects on increasing the quantity of ceftriaxone- and tetracycline-resistant *Salmonella* than the third-generation cephalosporin (CCFA) treatment itself.

### Quantitative dynamics of NTS *E*. *coli* in cattle treated with CCFA and CTC

**Fig 3:**
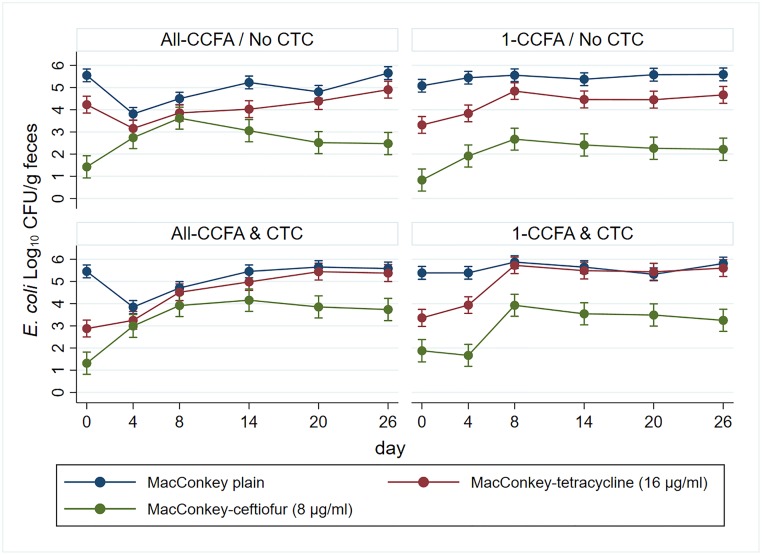
Quantity of *E*. *coli* was modeled by multilevel mixed linear regression. Blue: colony growth on plain MacConkey, red: colony growth on MacConkey-tetracycline, green: colony growth on MacConkey-ceftiofur.

Quantities (CFU) of NTS *E*. *coli* were also modeled using multilevel mixed linear regression and are presented in Fig 3. The NTS *E*. *coli* quantity decreased following CCFA treatment on Day 4 (Fig 3-left). In contrast to *Salmonella*, CTC treatment appeared to have no effect on the CFU counts of NTS *E*. *coli*. That said, the counts of tetracycline resistant *E*. *coli* were significantly increased by the CTC treatment (P<0.05). The differences in the counts between overall *E*. *coli* and tetracycline-resistant *E*. *coli* were reduced by the CTC treatment (Fig 3-bottom, blue and red line). Ceftiofur-resistant *E*. *coli* increased following the CCFA treatment. Even in the 1-CCFA & CTC groups, ceftiofur-resistant *E*. *coli* increased by Day 8 (Fig 3-right bottom).

#### Multilevel mixed-linear regression model of *invA* gene with representative imputed values

Among 1,040 samples tested by qPCR in duplicate (2,080 reactions), 571 reactions (27.5%) were detected as harboring the *invA* gene. The quantities of *invA* gene copies were standardized with the 16s rRNA gene by taking their ratio; subsequently, these were log_10_ transformed (Table 3, S4 Fig).

**Table 3:** Observed and 16s rRNA standardized log_10_ *invA* gene copies per gram feces per qPCR reaction.

*invA* gene detection	N	Mean (log_10_)	Median (log_10_)	Min (log_10_)	Max (log_10_)
Overall runs summary	571	4.43	4.32	2.78	8
Both duplicates detected	494	4.55	4.47	3.06	8
Missing in 1 reaction	77	3.65	3.65	2.78	4.58
16s standardized invA	N	Mean (log_10_)	Median (log_10_)	Min (log_10_)	Max (log_10_)
Overall runs summary	571	-5.13	-5.24	-7.2	-1.29
Both duplicates detected	494	-5	-5.08	-7.2	-1.29
Missing in 1 reaction	77	-5.95	-5.98	-7.03	-4.59

**Fig 4:**
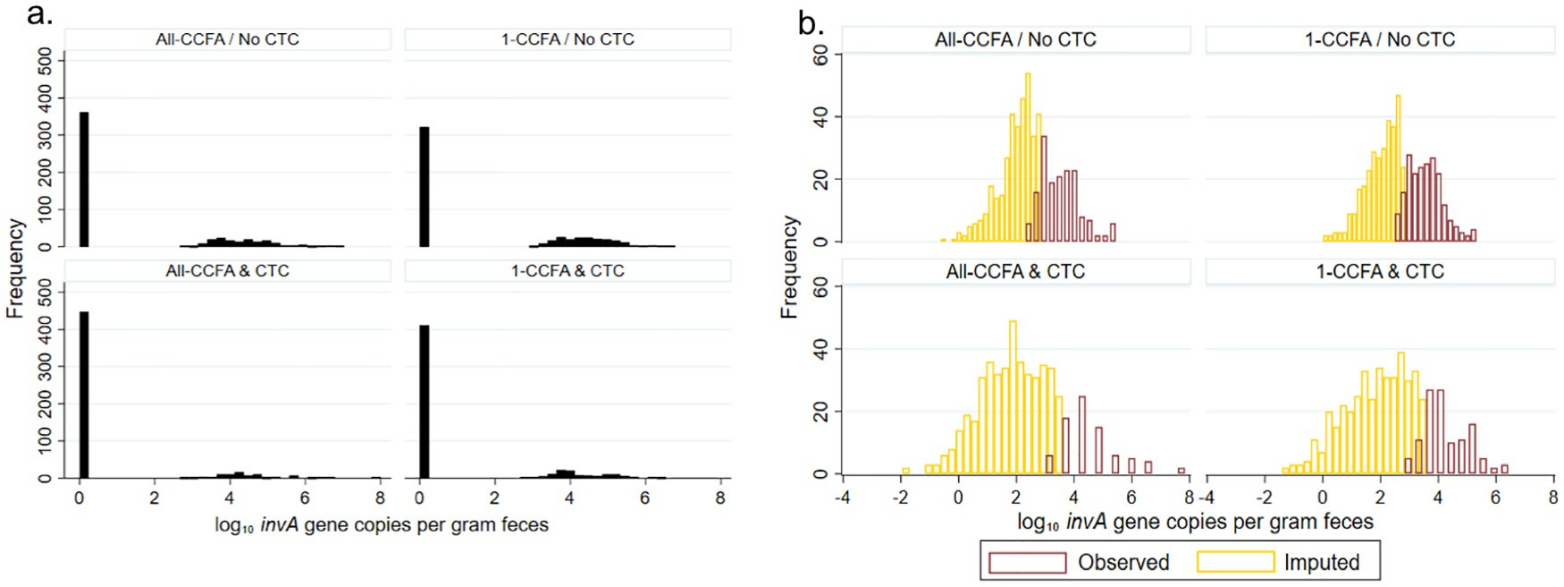
Distribution of log_10_ *invA* gene copies per gram feces per treatments before (a) and after imputation (b). Missing raw count below the limit of quantification were given a value of 1, which are shown as 0 following log_10_ transformation (a). CCFA: ceftiofur crystalline-free acid, CTC: chlortetracycline.

**Fig 5:**
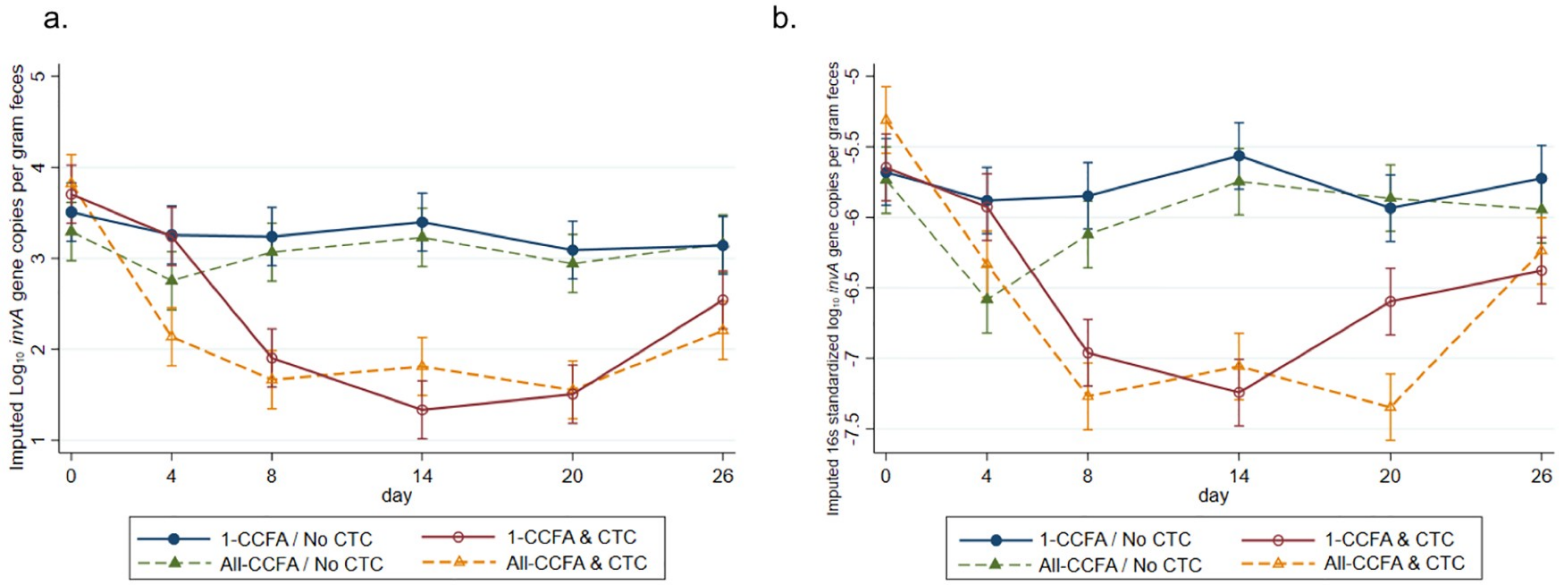
(a) Modeled marginal predictions with single imputed data for log_10_ *invA* gene copies per gram feces by treatments and days with 95% CI. (b) Modeled marginal predictions with single imputed data for 16s standardized log_10_ *invA* gene copies per gram feces by treatments and days with 95% CI. CCFA: ceftiofur crystalline-free acid, CTC: chlortetracycline.

Observations with missing values in 1,509 (72.5%) wells (i.e., those with Cq values > 40) from qPCR runs (shown in a bar as 0 in Fig 4(a)) were imputed using interval censored regression. The smallest imputed quantity after 20 imputation runs was as low as -1.95 log_10_ and the largest was 3.59 log_10_ (Fig 4(b), yellow bars). Marginal estimates of treatment effects on log_10_ *invA* gene copies per gram feces (Fig 5(a)) and those standardized with 16s rRNA (Fig 5(b)) were predicted by multilevel mixed linear regression. A significant decrease in imputed and observed gene copies was predicted for CTC treatment, especially on Day 8, where the animals were treated with only CTC (red) and showed a dramatic decrease. With CCFA treatment on Day 0, gene copies were also significantly decreased (orange). A slight increase in gene copies is observed following CTC treatment on Day 8 (orange) in the CCFA group. In the animals treated with CCFA on Day 0 (Green), but no CTC on Day8, only a slight decrease of gene copies was observed.

## Discussion

We demonstrated in our previous work that the overall prevalence of *Salmonella* (presence/absence, and by serotype) was greatly decreased by the two antibiotic treatments (CCFA and CTC) while the prevalence of MDR *Salmonella* increased in its place [27]. Here, we have demonstrated that the quantity of overall and MDR *Salmonella* follow a similar pattern to the prevalence dynamics. The risk of foodborne illness for pathogens such as *Salmonella* has been shown to be dose-dependent [22]. Thus, reduced quantity may also reduce risk of transmission through fecal contamination of either the environment or food. Resistant *Salmonella* became the dominant population following the antibiotic treatments, which also corresponds with our previous work [27]. Among populations of *E*. *coli*, tetracycline-resistant bacteria and ceftiofur-resistant bacteria behaved differently, where tetracycline-resistant *E*. *coli* expanded its quantity especially after CTC treatments, but ceftiofur-resistant *E*. *coli* did not increase in the presence of CTC, either subsequent to CCFA or alone.

### Effects of antibiotics on the quantity of *Salmonella*

Although previous mouse studies have shown that antibiotic treatments cause microbiota disruptions and increase *Salmonella* colonization [41], in the present study the quantity of *Salmonella* before (Day 0) and after (Day 26) were similar comparing antibiotic treated to untreated cattle.

In our study, roughly 15 cattle increased their *Salmonella* fecal quantity following the antibiotic treatments. Especially with CCFA treatment, roughly 5 cattle fecal CFU counts increased by 10 times through Day 26, though the sample size remains too small to derive conclusions. Not only antibiotics but also other factors such as feed contamination, high ambient temperatures, the manure-laden pen environment, and biting insects could affect *Salmonella* infection and quantities. Among the *Salmonella* that were detected in our study, resistant *Salmonella* have an advantage during and immediately following antibiotic exposure; as proof, tetracycline and ceftriaxone-resistant *Salmonella* were detected more frequently from those cattle and pens that were treated with CCFA and CTC.

It was expected that the antibiotic-resistant *Salmonella* population would increase in quantity following antibiotic treatment. However, no significant increases in quantity were observed after the antibiotic treatments (Fig 2). The quantities of tetracycline- and ceftriaxone-resistant *Salmonella* were relatively constant at around 4 log_10_ CFU/g feces. However, as the study period passed, more animals with growth on BGA-tet and BGA-cef were observed; indeed, this is very consistent with earlier prevalence results [27]. Much of the resistant *Salmonella* population appears to have resided below the LOQ before treatment, then increased to more than the LOQ, especially following CTC treatment.

### Comparison of *Salmonella* and NTS *E*. *coli* quantities

The quantity of NTS *E*. *coli* was suppressed by CCFA treatment but not by CTC treatment. On the other hand, the quantity of tetracycline-resistant *E*. *coli* increased almost to a quantity similar to total NTS *E*. *coli* following the CTC treatment. This suggests that tetracycline-resistant *E*. *coli* expanded and replaced the antibiotic-susceptible *E*. *coli* during the extended period of CTC treatment; therefore, the overall quantity of total NTS *E*. *coli* appeared unaffected by CTC. In contrast, for *Salmonella* the quantity was influenced by both CCFA and CTC treatment. CCFA treatment decreased the quantity and this was further suppressed by the CTC treatment. While tetracycline resistant *E*. *coli* dramatically replaced susceptible *E*. *coli*, the overall *Salmonella* population remained low after the CCFA treatment and was not replaced to the extent of *E*. *coli*. We can explain such differences between *Salmonella* and *E*. *coli* behavior by the vast differences of the quantity and genetic diversity and resistance pattern of the *E*. *coli* versus *Salmonella* populations. In previous work by Kanwar et al., which reported on the resistant *E*. *coli* population in the same samples, *E*. *coli* carrying *tet*(A) and *tet*(B) genes were commonly detected by PCR. *E*. *coli* with the *tet*(A) gene also were often carrying the *bla*_CMY-2_ gene, while *E*. *coli* detected with the *tet*(B) gene were less likely to be co-resistant with the *bla*_CMY-2_ gene [26]. NTS *E*. *coli* are known as a highly prevalent indicator bacteria and have more diverse genotypes as shown by PFGE and antibiotic resistance types in comparison to *Salmonella* serotypes, of which we identified only six [42].

Although we have not investigated the tetracycline resistance genes of the antibiotic-resistant *E*. *coli* that replaced the susceptible *E*. *coli*, *E*. *coli* with single tetracycline resistance may have a fitness advantage under CTC selection pressure. The resistance patterns of *Salmonella* detected from our prevalence study were more clonal, and most of the resistant isolates were either pan-susceptible or else of the MDR ampicillin-chloramphenicol-streptomycin-sulfisoxazol-tetracycline-ceftiofur (ACSSuT-Cef) phenotype [16]. In our previous work, prevalence of MDR *Salmonella* was 100% in the fecal samples that were tested after the CTC treatment on Day 14 and Day 20 [16]. It is likely that the MDR *Salmonella* were pre-selected by CCFA treatment and replaced the susceptible population with the subsequent CTC treatment. However, the quantity of *Salmonella* did not increase as dramatically as was seen in the *E*. *coli*. Even though the MDR *Salmonella* survived the antibiotic pressure, the fitness cost of resistance may not allow them to expand as dramatically.

Finally, we used ceftriaxone at the CLSI human clinical breakpoint of 4 μg/ml for spiral plating of *Salmonella* while we used the NARMS consensus breakpoint of 8 μg/ml for *E*. *coli*. We do not believe this has substantively affected the inference or comparisons that can be made from the data. Ceftriaxone is a common drug-of-choice for empirical therapy of human cases of salmonellosis. Ceftiofur is an animal-only drug and our interest was in exploring the dynamics of the indicator *E*. *coli* in response to that drug. The MIC patterns in in vitro assays, such as broth microdilution, for ceftiofur and ceftriaxone are virtually identical [26].

### Quantification by qPCR vs. direct spiral plating

Although *invA* gene copies and CFU of *Salmonella* per gram of feces are not directly comparable measurements, the LOQ of qPCR and direct plating to BGA was similar, around 2.6 log_10_ gene copies and CFU per gram of feces, respectively. However, among those samples detected positive by both qPCR and direct plating, the quantity of *Salmonella* was estimated at roughly 10 times higher in qPCR compared with direct plating. Because qPCR detects both viable and also non-viable *Salmonella* that might be killed by the antibiotics, it is possible that the quantity differences between qPCR and direct plating were simply due to the DNA arising from non-viable *Salmonella*. It is not likely that other bacterial species were detected with this *invA* gene unique to *Salmonella* [35].

In qPCR, multiple steps affect the quantity calculation, including efficiency of DNA extraction, type of template used for creating the standard curve, original fecal sample condition, and back calculation of gene copies to per gram of feces. We generated the standard curves from the whole-genome of *Salmonella* Typhimurium isolates, which had great reproducibility throughout the 30 individual qPCR 96-well plate runs. The total community DNA used in this study was extracted after the fecal samples were collected, having been stored at -80 °C for up to two years, and with the extracted DNA subsequently stored in the freezer at -20 °C for up to five years. On the other hand, the fecal samples that were used for the direct plating quantification had been stored at -80 °C with 50% glycerol for five years, which could weaken or degrade the bacteria and therefore decrease the counts. Based on our previous work on *Salmonella* prevalence, roughly 60% of samples that were detected with *Salmonella* through an enhanced enrichment process were quantifiable by either qPCR or direct spiral plating.

### Imputation of missing values

The qPCR results from our study contained many missing values (72.9% of the total wells failed to react using qPCR). This is problematic for data analysis due to bias produced because incomplete data will not be included in the analysis. In addition, multiple linear regression assumptions include normality of residuals which is problematic with zero-inflation of observations combined with normally distributed observed counts. To overcome this, we used multiple imputation for the analysis of treatment effects on the count of *invA* gene copies.

It has been shown that an appropriate imputation leads to less bias and better prediction of the model [43]. In our qPCR results, an interval censored regression model was chosen between linear regression and truncated regression because we expected that most of the values were missing due to being below the LOQ. Other methods impute values within the range of observed values which was clearly inappropriate for the situation in this study. Several possibilities for values below the LOQ can be considered; 1) cattle were not infected with *Salmonella* (true negative), 2) susceptible *Salmonella* were killed by antibiotic treatments or else starting quantities of viable *Salmonella* were very low, therefore Ct values were higher than 40 (censored data), 3) failure of qPCR runs due to PCR inhibitors, or 4) other reasons. Except for the first reason, the negative results can be interpreted as false negatives. It is highly probable that most samples belong to scenario 2), because, in the prevalence study, only 4 animals were not detected with any *Salmonella* across the 6 separate sampling days (representing up to 24 / 1040 samples) [16]. The model that was created after imputation was similar to that without imputation, but with predicted values much closer to the observed data (Fig 5). This shows that imputing the missing value can led to a less biased model. While few studies utilizing imputation methods to estimate the missing values in qPCR data have been reported, more established methods are clearly needed going forward [30, 43–45].

Until now, the risk of antibiotic use on the quantity of antibiotic-resistant *Salmonella* has not been well documented. In the current study, even though antibiotics led to a transient decrease in *Salmonella* quantity, more steers were shedding a quantifiable number of tetracycline- and ceftriaxone-resistant *Salmonella* by the end of the study. Additionally, among indicator *E*. *coli*, the susceptible population that was greatly reduced by antibiotics was quickly replaced by a tetracycline-resistant population. Antibiotic-resistant *Salmonella* contamination of meat products can pose substantial public health risks; therefore, further longer-term explorations of the risk of antibiotic use on the quantity of *Salmonella* up until the age of slaughter are warranted.

## Supporting information

S1 Fig Experimental design. Both replicates are combined. Treatment groups (boxes) represent a 2*2 factorial design of All-CCFA versus 1-CCFA and CTC versus no CTC. There were four pens per combined treatment group. Treatment with CCFA occurred on day 0 after the first fecal sample was taken, and was given to either all steers or else one steer in a pen. CTC was provided in three sequential 5-day pulses with a single day in between. Both treatment regimens were on label. Samples were collected every other day; however, only samples shown above (Days 0, 4, 8, 14, 20 and 26) were tested for *Salmonella* quantity.
(TIFF)

S2 Fig Venn diagram of sample numbers detected by different methods. **Left:** Number of samples detected with *Salmonella* by direct spiral plating and probe qPCR methods shown in a Venn diagram (out of 1,040 samples tested in total by each of the two methods). Gray: total samples tested, Green: detected via probe-based *invA* qPCR, Yellow: detected with direct spiral plating on plain BGA. **Right:** Growth of *Salmonella* on brilliant green agar (BGA), BGA-tetracycline (BGA-tet), and BGA-ceftriaxone (BGA-cef) from PBS diluted fecal samples. Yellow: detected with direct spiral plating on plain BGA, olive green: growth on BGA-cef, light blue: growth on BGA-tet. Numbers corresponds to the number of samples detected with *Salmonella* in each portion of the circle. The Venn-diagram was created with BioVenn [46].
(TIFF)

S3 Fig Observed quantity of susceptible, tetracycline, and ceftriaxone resistant *Salmonella* by antibiotic treatment and day with boxplot. Y-axis categories represent pen-level treatments. Treatment legend represents individual animal treatments within pen. Green: *invA* gene copies, Orange: Brilliant green agar (BGA) counts without antibiotics, Red: BGA with 4 μg/ml of ceftriaxone, Purple: BGA with 16 μg/ml of tetracycline. Boxplot represents the median and quartile range.
(TIFF)

S4 Fig Distribution of log_10_ *invA* gene copies from observed data. (a) Overall distribution of log_10_ *invA* gene copies per gram feces. Missing observations (below LOQ) were given a value 1 and log_10_ transformed to 0 in (a). (b) Distribution of log_10_ *invA* gene copies of samples that had none missing values (light blue) and missing 1 (green) in red squared area from (a). (c) Distribution of 16s rRNA standardized *invA* gene copies of samples that had none missing values (light blue) and missing 1 (green) in red squared area from (a). none missing:detected in duplicate wells, missing 1: detected in one well of the duplication.
(TIFF)

## References

[1] Scallan E, Hoekstra RM, Angulo FJ, Tauxe RV, Widdowson MA, Roy SL, et al Foodborne illness acquired in the United States—major pathogens. Emerg Infect Dis. 2011;17(1):7–15. 10.3201/eid1701.P11101 21192848 PMC3375761

[2] NARMS 2015 Integrated Report [Internet]. 2017. https://www.fda.gov/downloads/AnimalVeterinary/SafetyHealth/AntimicrobialResistance/NationalAntimicrobialResistanceMonitoringSystem/UCM581468.pdf.

[3] Pires SM, Vieira AR, Hald T, Cole D. Source attribution of human salmonellosis: an overview of methods and estimates. Foodborne Pathog Dis. 2014;11(9):667–76. Epub 2014/06/03. 10.1089/fpd.2014.1744 . 24885917 PMC10938214

[4] Clinton NA, Weaver RW, Hidalgo RJ. Transmission of Salmonella typhimurium among feedlot cattle after oral inoculation. The Journal of applied bacteriology. 1981;50(1):149–55. 10.1111/j.1365-2672.1981.tb00879.x . 7014545

[5] Dargatz DA, Strohmeyer RA, Morley PS, Hyatt DR, Salman MD. Characterization of Escherichia coli and Salmonella enterica from cattle feed ingredients. Foodborne Pathog Dis. 2005;2(4):341–7. 10.1089/fpd.2005.2.341 . 16366856

[6] Fegan N, Vanderlinde P, Higgs G, Desmarchelier P. Quantification and prevalence of Salmonella in beef cattle presenting at slaughter. J Appl Microbiol. 2004;97(5):892–8. 10.1111/j.1365-2672.2004.02380.x . 15479403

[7] Fegan N, Vanderlinde P, Higgs G, Desmarchelier P. A study of the prevalence and enumeration of Salmonella enterica in cattle and on carcasses during processing. J Food Prot. 2005;68(6):1147–53. Epub 2005/06/16. 10.4315/0362-028x-68.6.1147 . 15954700

[8] Brichta-Harhay DM, Arthur TM, Bosilevac JM, Guerini MN, Kalchayanand N, Koohmaraie M. Enumeration of Salmonella and Escherichia coli O157:H7 in ground beef, cattle carcass, hide and faecal samples using direct plating methods. J Appl Microbiol. 2007;103(5):1657–68. 10.1111/j.1365-2672.2007.03405.x . 17953577

[9] Kunze DJ, Loneragan GH, Platt TM, Miller MF, Besser TE, Koohmaraie M, et al Salmonella enterica burden in harvest-ready cattle populations from the southern high plains of the United States. Appl Environ Microbiol. 2008;74(2):345–51. 10.1128/AEM.02076-07 18024678 PMC2223257

[10] Arthur TM, Brichta-Harhay DM, Bosilevac JM, Kalchayanand N, Shackelford SD, Wheeler TL, et al Super shedding of Escherichia coli O157:H7 by cattle and the impact on beef carcass contamination. Meat Sci. 2010;86(1):32–7. 10.1016/j.meatsci.2010.04.019 . 20627603

[11] Wheeler TL, Kalchayanand N, Bosilevac JM. Pre- and post-harvest interventions to reduce pathogen contamination in the U.S. beef industry. Meat Sci. 2014;98(3):372–82. Epub 2014/07/17. 10.1016/j.meatsci.2014.06.026 . 25027798

[12] Brichta-Harhay DM, Arthur TM, Koohmaraie M. Enumeration of Salmonella from poultry carcass rinses via direct plating methods. Letters in applied microbiology. 2008;46(2):186–91. 10.1111/j.1472-765X.2007.02289.x . 18069983

[13] Koohmaraie M, Scanga JA, De La Zerda MJ, Koohmaraie B, Tapay L, Beskhlebnaya V, et al Tracking the sources of salmonella in ground beef produced from nonfed cattle. J Food Prot. 2012;75(8):1464–8. 10.4315/0362-028X.JFP-11-540 . 22856570

[14] Leekha S, Terrell CL, Edson RS. General principles of antimicrobial therapy. Mayo Clin Proc. 2011;86(2):156–67. Epub 2011/02/02. 10.4065/mcp.2010.0639 21282489 PMC3031442

[15] FDA. Summary Report on Antimicrobials Sold or Distributed for Use in Food-Producing Animals 2015

[16] Ohta N, Norman KN, Norby B, Lawhon SD, Vinasco J, den Bakker H, et al Population dynamics of enteric Salmonella in response to antimicrobial use in beef feedlot cattle. Sci Rep. 2017;7(1):14310 10.1038/s41598-017-14751-9 29085049 PMC5662634

[17] Singer RS, Patterson SK, Wallace RL. Effects of therapeutic ceftiofur administration to dairy cattle on Escherichia coli dynamics in the intestinal tract. Appl Environ Microbiol. 2008;74(22):6956–62. 10.1128/AEM.01241-08 18820057 PMC2583494

[18] Daniels JB, Call DR, Hancock D, Sischo WM, Baker K, Besser TE. Role of ceftiofur in selection and dissemination of blaCMY-2-mediated cephalosporin resistance in Salmonella enterica and commensal Escherichia coli isolates from cattle. Appl Environ Microbiol. 2009;75(11):3648–55. 10.1128/AEM.02435-08 19376926 PMC2687309

[19] Schmidt JW, Griffin D, Kuehn LA, Brichta-Harhay DM. Influence of therapeutic ceftiofur treatments of feedlot cattle on fecal and hide prevalences of commensal Escherichia coli resistant to expanded-spectrum cephalosporins, and molecular characterization of resistant isolates. Appl Environ Microbiol. 2013;79(7):2273–83. 10.1128/AEM.03592-12 23354706 PMC3623230

[20] Agga GE, Schmidt JW, Arthur TM. Effects of In-Feed Chlortetracycline Prophylaxis in Beef Cattle on Animal Health and Antimicrobial-Resistant Escherichia coli. Appl Environ Microbiol. 2016;82(24):7197–204. 10.1128/AEM.01928-16 27736789 PMC5118930

[21] Cassin MH, Lammerding AM, Todd EC, Ross W, McColl RS. Quantitative risk assessment for Escherichia coli O157:H7 in ground beef hamburgers. International journal of food microbiology. 1998;41(1):21–44. Epub 1998/06/19. 10.1016/s0168-1605(98)00028-2 . 9631335

[22] McCullough NB, Eisele CW. Experimental human salmonellosis. I. Pathogenicity of strains of Salmonella meleagridis and Salmonella anatum obtained from spray-dried whole egg. The Journal of infectious diseases. 1951;88(3):278–89. Epub 1951/05/01. 10.1093/infdis/88.3.278 . 14850755

[23] Mirzaagha P, Louie M, Sharma R, Yanke LJ, Topp E, McAllister TA. Distribution and characterization of ampicillin- and tetracycline-resistant Escherichia coli from feedlot cattle fed subtherapeutic antimicrobials. BMC Microbiol. 2011;11:78 10.1186/1471-2180-11-78 . 21504594 PMC3103423

[24] Kadykalo SV, Anderson MEC, Alsop JE. Passive surveillance of antimicrobial resistance in Salmonella and Escherichia coli isolates from Ontario livestock, 2007–2015. The Canadian veterinary journal La revue veterinaire canadienne. 2018;59(6):617–22. Epub 2018/06/19. 29910475 PMC5949941

[25] Douard G, Praud K, Cloeckaert A, Doublet B. The Salmonella genomic island 1 is specifically mobilized in trans by the IncA/C multidrug resistance plasmid family. PLoS One. 2010;5(12):e15302 10.1371/journal.pone.0015302 21187963 PMC3004903

[26] Kanwar N, Scott HM, Norby B, Loneragan GH, Vinasco J, McGowan M, et al Effects of ceftiofur and chlortetracycline treatment strategies on antimicrobial susceptibility and on tet(A), tet(B), and bla CMY-2 resistance genes among E. coli isolated from the feces of feedlot cattle. PloS one. 2013;8(11):e80575 10.1371/journal.pone.0080575 24260423 PMC3834275

[27] CLSI. Performance standards for antimicrobial disk susceptibility tests; Approved standard-Twelfth Edition Clinical and Laboratory Standards Institute, Wayne, PA 2015.

[28] NARMS. Antibiotics tested by NARMS [updated March 15, 2019]. https://www.cdc.gov/narms/antibiotics-tested.html.

[29] Lowrance TC, Loneragan GH, Kunze DJ, Platt TM, Ives SE, Scott HM, et al Changes in antimicrobial susceptibility in a population of Escherichia coli isolated from feedlot cattle administered ceftiofur crystalline-free acid. American Journal of Veterinary Research. 2007;68(5):501–7. 10.2460/ajvr.68.5.501 17472449

[30] Kanwar N, Scott HM, Norby B, Loneragan GH, Vinasco J, Cottell JL, et al Impact of treatment strategies on cephalosporin and tetracycline resistance gene quantities in the bovine fecal metagenome. Scientific reports. 2014;4:5100 10.1038/srep05100 24872333 PMC5381505

[31] Fey A, Eichler S, Flavier S, Christen R, Hofle MG, Guzman CA. Establishment of a real-time PCR-based approach for accurate quantification of bacterial RNA targets in water, using Salmonella as a model organism. Appl Environ Microbiol. 2004;70(6):3618–23. 10.1128/AEM.70.6.3618-3623.2004 15184165 PMC427797

[32] Brankatschk R, Bodenhausen N, Zeyer J, Burgmann H. Simple absolute quantification method correcting for quantitative PCR efficiency variations for microbial community samples. Appl Environ Microbiol. 2012;78(12):4481–9. Epub 2012/04/12. 10.1128/AEM.07878-11 22492459 PMC3370567

[33] Galan JE, Ginocchio C, Costeas P. Molecular and functional characterization of the Salmonella invasion gene invA: homology of InvA to members of a new protein family. J Bacteriol. 1992;174(13):4338–49. Epub 1992/07/01. 10.1128/jb.174.13.4338-4349.1992 1624429 PMC206218

[34] Rahn K, De Grandis SA, Clarke RC, McEwen SA, Galan JE, Ginocchio C, et al Amplification of an invA gene sequence of Salmonella typhimurium by polymerase chain reaction as a specific method of detection of Salmonella. Molecular and cellular probes. 1992;6(4):271–9. Epub 1992/08/01. 10.1016/0890-8508(92)90002-f . 1528198

[35] Gonzalez-Escalona N, Hammack TS, Russell M, Jacobson AP, De Jesus AJ, Brown EW, et al Detection of live Salmonella sp. cells in produce by a TaqMan-based quantitative reverse transcriptase real-time PCR targeting invA mRNA. Appl Environ Microbiol. 2009;75(11):3714–20. 10.1128/AEM.02686-08 19376910 PMC2687310

[36] Steinman CR, Muralidhar B, Nuovo GJ, Rumore PM, Yu D, Mukai M. Domain-directed polymerase chain reaction capable of distinguishing bacterial from host DNA at the single-cell level: characterization of a systematic method to investigate putative bacterial infection in idiopathic disease. Anal Biochem. 1997;244(2):328–39. Epub 1997/01/15. 10.1006/abio.1996.9896 . 9025950

[37] Pires AF, Funk JA, Lim A, Bolin SR. Enumeration of salmonella in feces of naturally infected pigs. Foodborne Pathog Dis. 2013;10(11):933–7. 10.1089/fpd.2013.1547 . 23944750

[38] Bustin SA, Benes V, Garson JA, Hellemans J, Huggett J, Kubista M, et al The MIQE guidelines: minimum information for publication of quantitative real-time PCR experiments. Clin Chem. 2009;55(4):611–22. Epub 2009/02/28. 10.1373/clinchem.2008.112797 . 19246619

[39] Y. M. Multiple-imputation analysis using Stata’s mi command. 2010 [cited 2018 3-29-2018]. https://www.stata.com/meeting/boston10/boston10_marchenko.pdf.

[40] Yulia V. Marchenko WE. A note on how to perform multiple-imputation diagnostics in Stata [cited 2018 3-29-2018]. https://www.stata.com/users/ymarchenko/midiagnote.pdf.

[41] Ng KM, Ferreyra JA, Higginbottom SK, Lynch JB, Kashyap PC, Gopinath S, et al Microbiota-liberated host sugars facilitate post-antibiotic expansion of enteric pathogens. Nature. 2013;502(7469):96–9. 10.1038/nature12503 23995682 PMC3825626

[42] Schmidt JW, Agga GE, Bosilevac JM, Brichta-Harhay DM, Shackelford SD, Wang R, et al Occurrence of Antimicrobial-Resistant Escherichia coli and Salmonella enterica in the Beef Cattle Production and Processing Continuum. Appl Environ Microbiol. 2015;81(2):713–25. 10.1128/AEM.03079-14 25398858 PMC4277590

[43] McCall MN, McMurray HR, Land H, Almudevar A. On non-detects in qPCR data. Bioinformatics. 2014;30(16):2310–6. Epub 2014/04/26. 10.1093/bioinformatics/btu239 24764462 PMC4133581

[44] Sterne JA, White IR, Carlin JB, Spratt M, Royston P, Kenward MG, et al Multiple imputation for missing data in epidemiological and clinical research: potential and pitfalls. Bmj. 2009;338:b2393 Epub 2009/07/01. 10.1136/bmj.b2393 19564179 PMC2714692

[45] Boyer TC, Hanson T, Singer RS. Estimation of low quantity genes: a hierarchical model for analyzing censored quantitative real-time PCR data. PLoS One. 2013;8(5):e64900 Epub 2013/06/07. 10.1371/journal.pone.0064900 23741414 PMC3669010

[46] Hulsen T, de Vlieg J, Alkema W. BioVenn—a web application for the comparison and visualization of biological lists using area-proportional Venn diagrams. BMC genomics. 2008;9:488 Epub 2008/10/18. 10.1186/1471-2164-9-488 18925949 PMC2584113

