# Supplementary figures and images for: Quantitative dynamics of Salmonella and E. coli in feces of feedlot cattle treated with ceftiofur and chlortetracycline
Source: PLoS One. 2019 Dec 2;14(12):e0225697. doi: 10.1371/journal.pone.0225697 (PMC6887520; doi:10.1371/journal.pone.0225697)

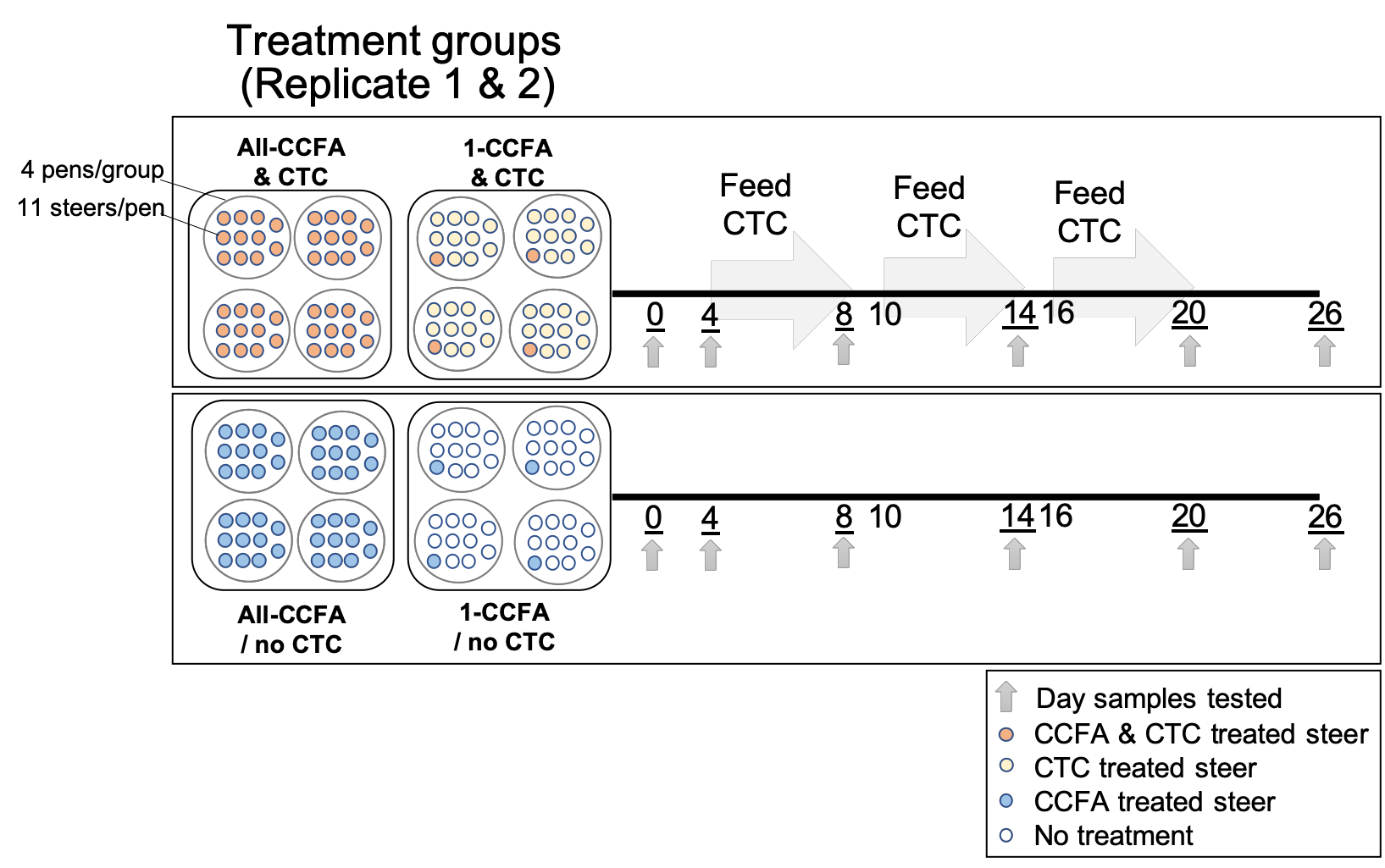

Supplement: S1 Fig — Both replicates are combined. Treatment groups (boxes) represent a 2*2 factorial design of All-CCFA versus 1-CCFA and CTC versus no CTC. There were four pens per combined treatment group. Treatment with CCFA occurred on day 0 after the first fecal sample was taken, and was given to either all steers or else one steer in a pen. CTC was provided in three sequential 5-day pulses with a single day in between. Both treatment regimens were on label. Samples were collected every other day; however, only samples shown above (Days 0, 4, 8, 14, 20 and 26) were tested for Salmonella quantity. (TIFF) [file pone.0225697.s001.tiff]

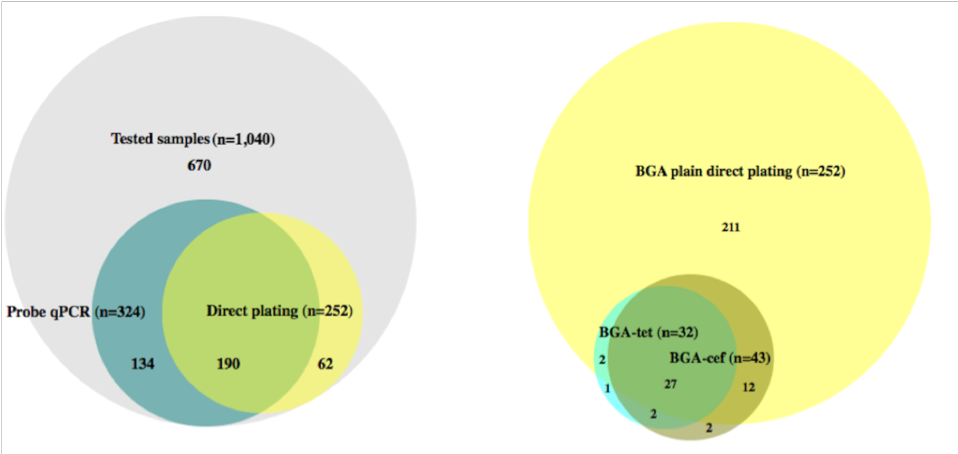

Supplement: S2 Fig — Left: Number of samples detected with Salmonella by direct spiral plating and probe qPCR methods shown in a Venn diagram (out of 1,040 samples tested in total by each of the two methods). Gray: total samples tested, Green: detected via probe-based invA qPCR, Yellow: detected with direct spiral plating on plain BGA. Right: Growth of Salmonella on brilliant green agar (BGA), BGA-tetracycline (BGA-tet), and BGA-ceftriaxone (BGA-cef) from PBS diluted fecal samples. Yellow: detected with direct spiral plating on plain BGA, olive green: growth on BGA-cef, light blue: growth on BGA-tet. Numbers corresponds to the number of samples detected with Salmonella in each portion of the circle. The Venn-diagram was created with BioVenn [46]. (TIFF) [file pone.0225697.s002.tiff]

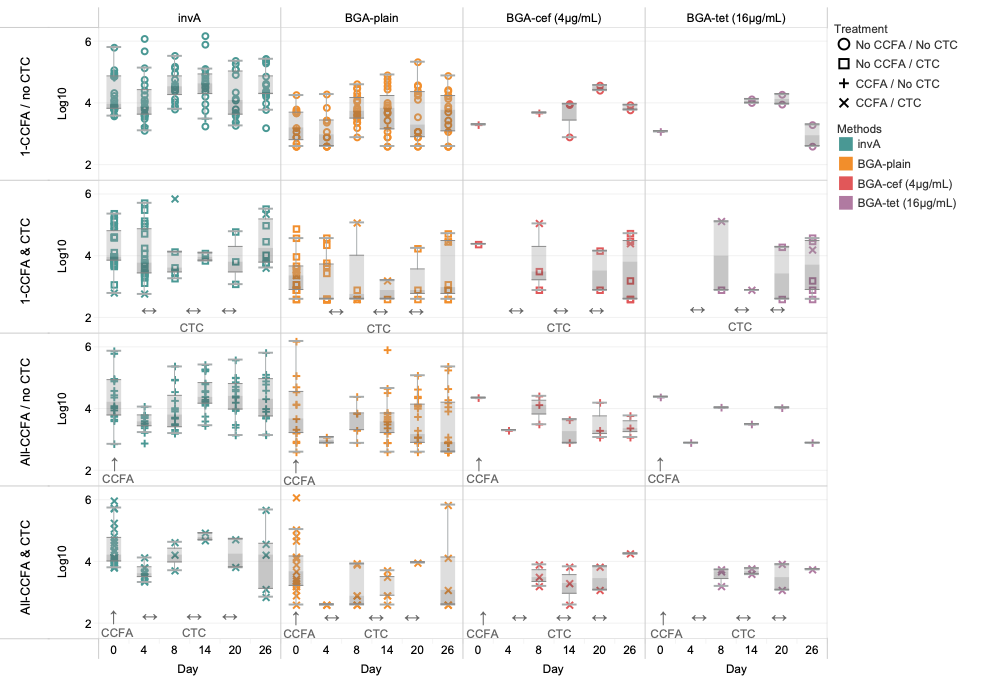

Supplement: S3 Fig — Y-axis categories represent pen-level treatments. Treatment legend represents individual animal treatments within pen. Green: invA gene copies, Orange: Brilliant green agar (BGA) counts without antibiotics, Red: BGA with 4 μg/ml of ceftriaxone, Purple: BGA with 16 μg/ml of tetracycline. Boxplot represents the median and quartile range. (TIFF) [file pone.0225697.s003.tiff]

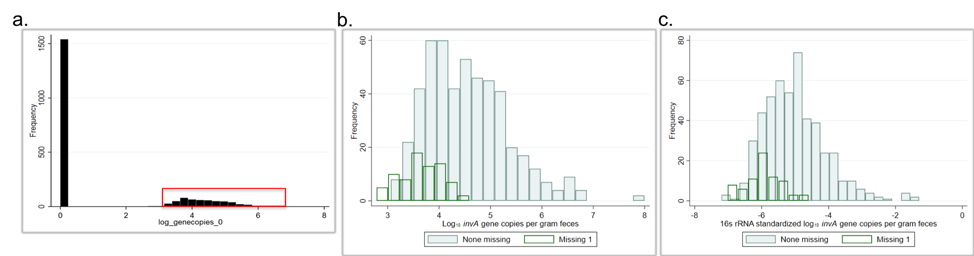

Supplement: S4 Fig — (a) Overall distribution of log10 invA gene copies per gram feces. Missing observations (below LOQ) were given a value 1 and log10 transformed to 0 in (a). (b) Distribution of log10 invA gene copies of samples that had none missing values (light blue) and missing 1 (green) in red squared area from (a). (c) Distribution of 16s rRNA standardized invA gene copies of samples that had none missing values (light blue) and missing 1 (green) in red squared area from (a). none missing:detected in duplicate wells, missing 1: detected in one well of the duplication. (TIFF) [file pone.0225697.s004.tiff]
